# The causal effects of age at menarche and age at menopause on sepsis: A two-sample Mendelian randomization analysis

**DOI:** 10.1371/journal.pone.0293540

**Published:** 2024-02-07

**Authors:** Na Guo, Lu Zhang, Nannan He, Hong Guo, Jian Liu

**Affiliations:** 1 The Fist Clinical Medical College of Lanzhou University, Lanzhou, Gansu Province, China; 2 Department of Intensive Care Unit, The First Hospital of Lanzhou University, Lan Zhou, Gansu Province, China; 3 Department of Intensive Care Unit, Gansu Provincial Maternity and Child Health Hospital/Gansu Provincial General Hospital, Lan Zhou, Gansu Province, China; Jinan University First Affiliated Hospital, CHINA

## Abstract

**Objectives:**

To determine whether the age at menarche (AAM) and the age at menopause (ANM) are causally related to the development of sepsis.

**Methods:**

We performed a two-sample Mendelian randomization (MR) analysis by utilizing summary statistics from genome-wide association study (GWAS) datasets for both the exposure and outcome variables. Single nucleotide polymorphisms (SNPs) that exhibited significant associations with AAM and ANM were chosen as instrumental variables to estimate the causal effects on sepsis. Our study employed a variety of methods, including MR-Egger regression, weighted median estimation, inverse variance weighting, a simple model, and a weighted model. Odds ratios (ORs) along with their corresponding 95% confidence intervals (CIs) were used as the primary indicators for assessing causality. Furthermore, we conducted sensitivity analyses to explore the presence of genetic heterogeneity and validate the robustness of the tools employed.

**Result:**

Our analysis revealed a significant negative causal relationship between AAM and the risk of sepsis (IVW: OR = 0.870, 95% CI = 0.793–0.955, *P* = 0.003). However, our Mendelian randomization (MR) analysis did not yield sufficient evidence to support a causal link between ANM and sepsis (IVW: OR = 0.987, 95% CI = 0.971–1.004, *P* = 0.129).

**Conclusions:**

Our findings suggest that an earlier AAM may be associated with an increased risk of sepsis. However, we did not find sufficient evidence to support a causal relationship between ANM and sepsis.

## Introduction

Sepsis is a life-threatening condition characterized by organ dysfunction, arising from the dysregulation of the body’s immune response, often linked to infectious diseases [1]. Globally, sepsis is estimated to afflict around 30 million individuals annually, re sulting in six million fatalities [2]. Despite advances in early goal-directed therapy and tailored treatment approaches, sepsis continues to impose a substantial burden on global health, prompting the World Health Organization to designate it as a critical public health concern. Therefore, the pursuit of therapeutic and preventive targets to enhance sepsis outcomes remains of paramount importance.

Female reproductive factors, particularly age at menarche and age at menopause, are genetically influenced traits that exhibit considerable interindividual variation. These factors have been associated with a wide range of conditions, including lung cancer [3], depression [4], osteoporosis [5], Parkinson’s disease [6], and cardiovascular disease [7, 8]. Understanding the impact of age at menarche and age at menopause offers insights into the pathophysiology of these diseases, shedding light on the potential repercussions of early or late exposure to sex hormones on women’s health outcomes and contributing to our comprehension of gender-specific disparities in common disease risks [9–11]. Epidemiological investigations have indicated that gender plays a pivotal role in the occurrence and prognosis of sepsis syndromes. Although sepsis incidence appears higher in males [12], certain studies have surprisingly suggested that sepsis mortality rates may be elevated in females [13, 14]. The underlying mechanisms responsible for these epidemiological distinctions remain elusive. Substantial evidence indicates that sepsis has been linked to irregular menstruation, the menopausal transition, and both natural and surgical menopause, with reports of sepsis improvement in postmenopausal women receiving hormone replacement therapy [15]. However, a causal connection between reproductive factors and sepsis risk has not been conclusively established.

The MR method has emerged as a valuable tool for deducing causal relationships between exposures and outcomes, employing SNPs as IVs [16]. These variants are randomly assigned to offspring at conception, akin to the random allocation in randomized controlled trials (RCTs), thereby mitigating the influence of confounding variables and reverse causality [17, 18]. In this study, we conducted a two-sample MR analysis with the objective of exploring the causal link between AAM and ANM and the risk of sepsis in women. MR represents a novel epidemiological approach that has gained substantial traction in recent decades, finding application across various research domains. To the best of our knowledge, no prior investigation has probed the causal nexus between reproductive factors and sepsis. Consequently, our study pioneers the utilization of MR analysis, harnessing genetic variability to unveil the causal connection between female reproductive factors and sepsis risk.

## Materials and methods

### Study design

**Fig 1:**
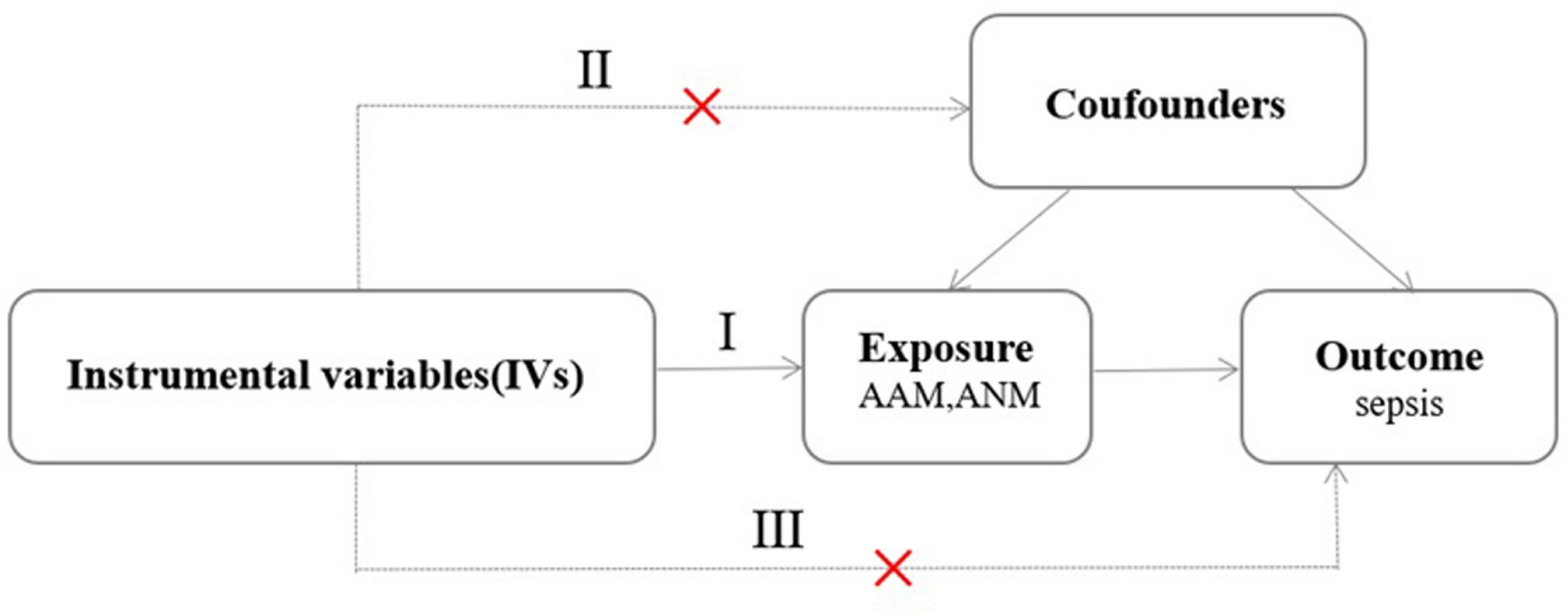
Schematic depicting three key assumptions: (I) identified SNPs must be highly correlated with AAM and ANM; (II) SNPs should be independent of any confounding variables; and (III) SNPs affect the results only through their effect on exposure.

This study employed a two-sample MR analysis conducted using SNPs derived from GWAS summary data. The exposure variables of interest were AAM and ANM, while the outcome was sepsis. To establish a robust MR framework, we formulated the following hypotheses (Fig 1): (I) the chosen SNPs must exhibit a strong correlation with the investigated exposure variables; (II) these SNPs should be free from associations with any confounding variables; and (III) the impact of these SNPs on the outcomes is solely mediated through the exposure variables.

### Data sources

Sepsis-related data were obtained from the UK Biobank, comprising a cohort of 486,484 European participants [19]. GWAS summary data for age at menarche (n = 182,416) [20] and age at natural menopause (n = 69,360) [21] were generously provided by the Reproductive Genetics (ReproGen) Consortium. It is noteworthy that all GWAS datasets were derived from independent cohorts of European ancestry, as detailed in S1 Table. Importantly, for the purposes of our analysis, we utilized these publicly available datasets without necessitating additional ethical approval.

### SNP selection

Given the pivotal role of MR analysis in studying causality, our study employed this approach to investigate the potential influence of AAM and ANM on sepsis development. SNPs associated with AAM and ANM were utilized as IVs in the MR analysis. To ensure the robustness of our IVs, we adhered to stringent criteria. All identified SNPs were required to exhibit a strong correlation with the respective exposure factors (*P*<5×10^−8^) and maintain a linkage disequilibrium (LD) r^2^ value of less than 0.01 within a 10,000 kb window [22]. The effectiveness of each genetic instrument was evaluated using the F statistic (F = R^2^/(1-R^2^)×[(N-K-1)/K]), where R^2^ represents the proportion of exposure variance explained by the chosen IV, N represents the sample size, and K represents the number of SNPs [18]. Specifically, the F statistic was computed using the formula 2×beta^2^×(1-EAF)×EAF, where EAF signifies the effect allele frequency, and beta denotes the allele effect value [23]. SNPs with F statistics below 10 were categorized as weak instruments and were consequently excluded from the MR analysis [24]. Additionally, we excluded SNPs with palindromic or incompatible alleles when harmonizing exposure and outcome variables. Ultimately, our analysis included 61 independent AAM SNPs and 166 independent ANM SNPs as instrumental variables. Detailed information on the SNPs employed as instrumental variables can be found in S2 and S3 Tables.

### Statistical analysis

To evaluate the causal impact of exposure factors on sepsis, we employed five distinct statistical approaches, namely MR-Egger [25], IVW [26], weighted median [27], simple model, and weighted model. Among these, IVW is widely recognized as the most reliable MR method. Initially, we utilized MR-PRESSO to identify potential outliers. In cases where outliers were identified, they were excluded from the analysis, and the assessment was re-executed. Subsequently, we conducted a leave-one-out sensitivity analysis, where each SNP was sequentially omitted, and the remaining SNPs were subjected to IVW analysis. This process enabled us to gauge the individual SNP’s impact on IVW results.Furthermore, we employed the MR-Egger method [28] to assess horizontal pleiotropy, with a P-value <0.05 indicating the presence of horizontal pleiotropy [29]. Additionally, Cochran’s Q test was applied to evaluate heterogeneity among instrumental variables, while a funnel plot was used to scrutinize potential bias in the study outcomes.

All statistical analyses were conducted using R statistical software (version 4.3.0) and the "TwoSample MR" package (version 0.5.6). We considered a significance level of P<0.05 as indicative of statistical significance. Given that the outcome variable is binary and categorical, we further converted the estimated effects into ORs to provide a more intuitive assessment of the relationship between AAM, ANM, and sepsis.

## Results

### Selection of instrumental variables

Following the exclusion of SNPs with palindromic alleles, low allele frequencies, weak instrumental characteristics, and SNPs explaining more outcome variance than the variance of the respective exposure factor, we identified a total of 61 SNPs associated with AAM and 166 SNPs linked to ANM for subsequent MR analysis. Notably, all of these SNPs exhibited F statistics exceeding 10, thereby meeting the stringent criteria for strong instrumental correlation assumption, as mandated in MR studies.

### The causal effect of age at menarche on sepsis

**Fig 2:**
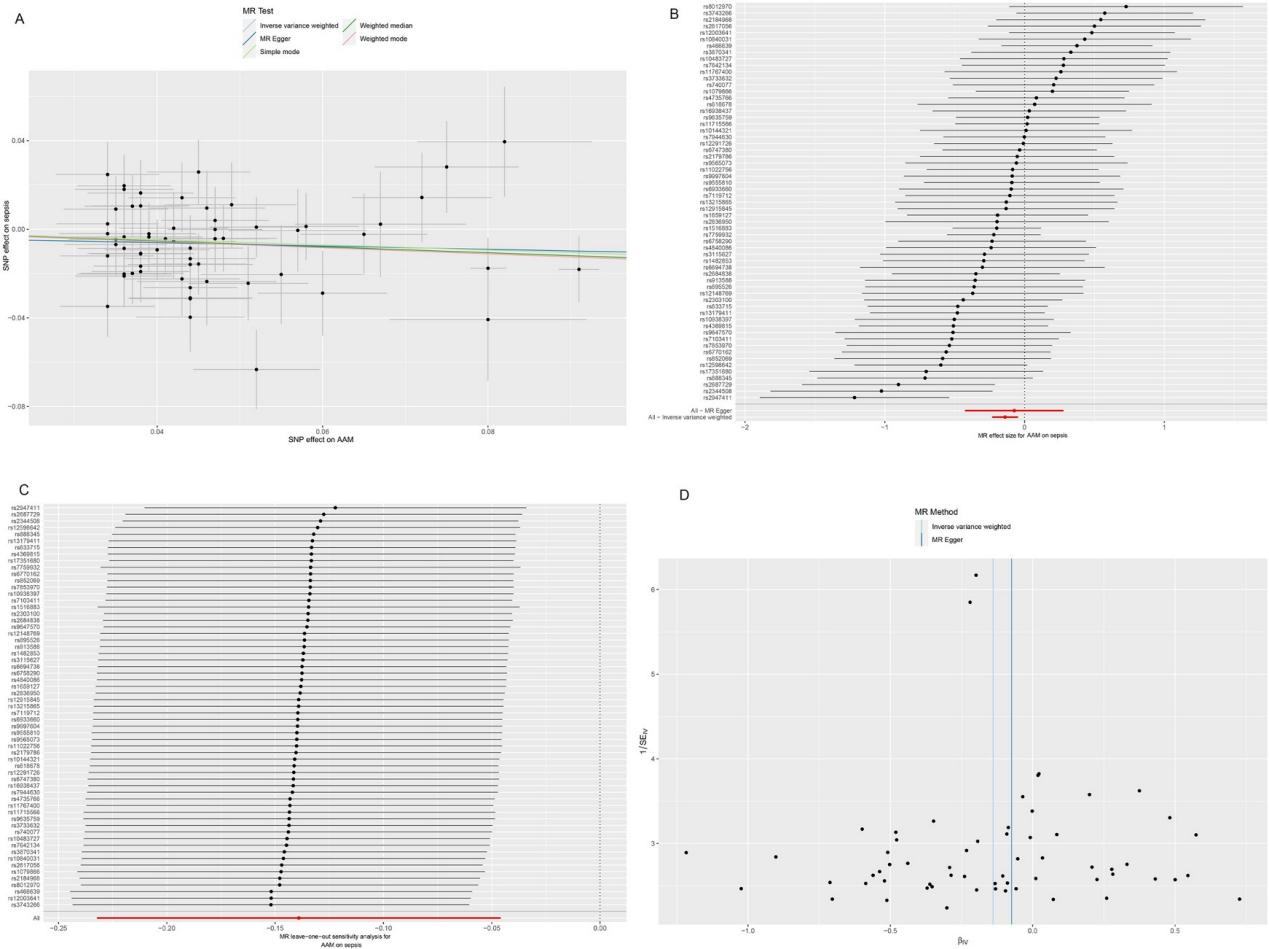
Scatter plot (A), forest plot (B), sensitivity analysis (C), and funnel plot (D) of the causal effect of AAM on sepsis. AAM: age at menarche.

In our causal analysis employing the Inverse Variance Weighting (IVW) method, the results provided substantial evidence supporting a negative causal association between AAM and the risk of sepsis, with an OR of 0.870 (95% CI = 0.793–0.955, *P* = 0.003). In sensitivity analyses, Cochran’s Q test revealed no significant heterogeneity (Cochran’s Q Statistic = 73.567, *P* = 0.112), while MR-Egger regression analysis indicated no indication of multiplicity (intercept = −0.003; *P* = 0.708) (Table 1). The scatter plot (Fig 2A) provides a graphical representation of the causal relationship between AAM and the risk of sepsis, while the forest plot (Fig 2B) visually depicts the causal effect of AAM on sepsis risk. Leave-one-out sensitivity analyses, which involved the sequential exclusion of each of the 61 SNPs associated with sepsis, demonstrated no significant alterations in the results (all data points remained consistently on the same side of 0). This observation underscores the reliability of the MR analysis outcomes (Fig 2C).

**Table 1:** Results of the MR analysis.

Outcome	Methods	SNP	OR(95CI)	SE	*P*-value	Cochran’s Q statistic (p-value)	Egger intercept (pvalue)
AAM	MR-egger	61	0.929(0.653–1.321)	0.180	0.683	73.391(0.098)	-0.003(0.708)
	Weighted median	61	0.876(0.772–0.995)	0.065	0.041		
	Inverse variance Weighted	61	0.870(0.793–0.955)	0.048	0.003	73.567(0.112)	
	Simple mode	61	0.890(0.662–1.195)	0.151	0.441		
	Weighted mode	61	0.870(0.705–1.074)	0.107	0.201		
ANM	MR-egger	166	0.994(0.964–1.025)	0.016	0.692	161.707(0.536)	-0.001(0.618)
	Weighted median	166	1.007(0.979–1.036)	0.014	0.619		
	Inverse variance Weighted	166	0.987(0.971–1.004)	0.008	0.129	161.956(0.552)	
	Simple mode	166	1.009(0.949–1.072)	0.030	0.778		
	Weighted mode	166	1.004(0.974–1.036)	0.016	0.779		

AAM: age at menarche; ANM: age at menopause.

### The causal effect of age at menopause on sepsis

**Fig 3:**
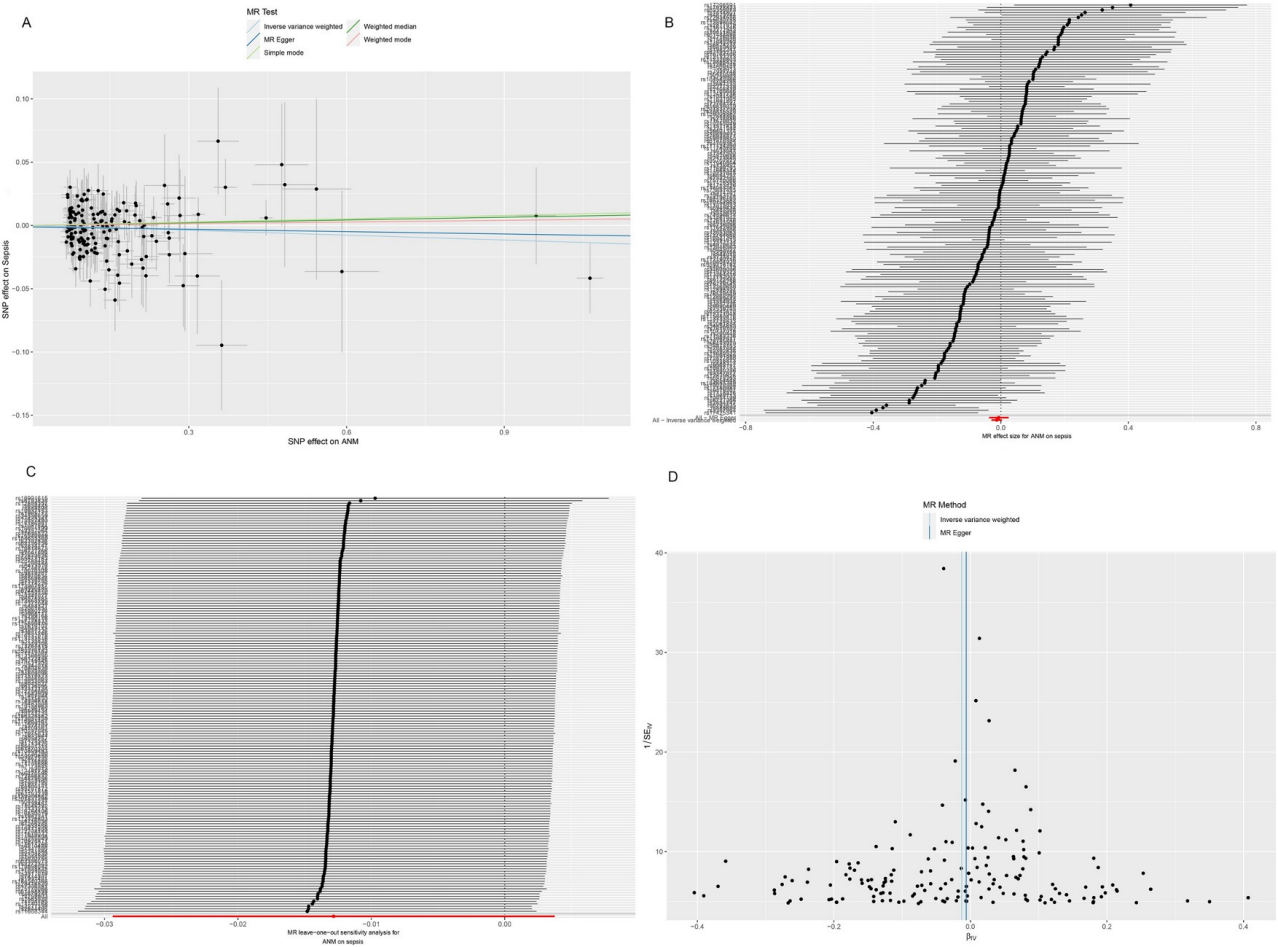
Scatter plot (A), forest plot (B), sensitivity analysis (C), and funnel plot (D) of the causal effect of ANM on sepsis. ANM: age at menopause.

MR analyses performed on both datasets yielded results indicating the absence of a causal relationship between age at menopause and the risk of sepsis (IVW method: OR = 0.987, 95% CI = 0.971–1.004, *P* = 0.129). Furthermore, our analysis revealed no significant heterogeneity (Cochran’s Q statistic = 161.956, *P* = 0.552), and no evidence of pleiotropy was detected (MR-Egger intercept = -0.001, *P* = 0.618) (Table 1 and Fig 3).

## Discussion

In our study, we investigated the potential causal relationship between reproductive factors and the risk of sepsis using a two-sample MR analysis. Our findings revealed a significant negative causal association between AAM and sepsis, indicating that early menarche may elevate the risk of sepsis development. However, we did not identify a clear link between ANM and sepsis. These results contribute valuable insights into the underlying biological mechanisms of sepsis and hint at AAM as a potential risk factor for sepsis, suggesting that women who experience early menarche may require closer monitoring for sepsis.

Prior research has suggested that females may have a reduced susceptibility to sepsis, possibly due to the protective effects of estrogen [30–32]. Epidemiological studies have increasingly examined the impact of gender on sepsis incidence and prevalence, with men being more susceptible to sepsis than women. Observational studies have reported higher sepsis incidence and organ dysfunction in men compared to women with similar injury scores [33]. Another study in a general intensive care unit context found that men were more likely to develop sepsis on admission and progress to sepsis and organ failure during their stay [34]. However, a causal link between reproductive factors and sepsis had not been previously established. Our study suggests that early menarche may indeed be causally associated with an increased sepsis risk. Several reasons support this conclusion: (1) Research by Zhang et al. [35] indicates that early menarche is associated with higher C-reactive protein (CRP) levels. (2) Early menarche might render women more susceptible to life-related stresses, potentially leading to heightened sympathetic nervous system activity [36]. (3) Early menarche can result in early estrogen exposure, elevated estradiol levels, and potential adverse effects on vascular elasticity and coagulation [37, 38]. (4) Premature estrogen exposure due to early menarche could lead to significant estrogen accumulation effects. As socioeconomic conditions improve, the age of menarche among females is trending earlier [39], potentially elevating sepsis risk in the female population.

Menopause in women signifies a loss of fertility and a significant decline in endogenous estrogen production, strongly linked to various adverse health outcomes. Intriguingly, our study did not establish a causal relationship between ANM and sepsis prevalence, contrary to several previous studies. These earlier investigations indicated that women under the age of 50 have a significantly lower likelihood of developing sepsis [40] and a reduced risk of post-trauma mortality compared to men [41, 42], though this effect diminishes after age 50 [43, 44]. Additionally, hormone-regulating medications like postmenopausal hormone therapy have been associated with reduced sepsis risk [15].

This MR study is the first to explore the association of AAM and ANM with sepsis risk, offering unique advantages over traditional observational studies, particularly in minimizing residual confounding factors. Thus, our findings contribute novel insights into sex-related disparities in sepsis incidence. We employed data from a recently published large-scale GWAS and conducted multiple sensitivity analyses to validate the robustness of our results.

Nonetheless, our study has limitations. Firstly, since our analyses focused solely on European populations, the generalizability of our results to other ethnic groups may be limited, warranting caution when extrapolating findings to other populations. Secondly, despite our efforts to identify potential surrogate SNPs, not all relevant exposure SNPs were available in the resulting GWAS dataset. Consequently, many exposure SNPs were not included in our MR analysis. While this may have affected the statistical power to detect small effects, we incorporated a substantial number of SNPs and conducted a rigorous MR analysis. Thirdly, our use of a sepsis-related dataset that included both males and females could potentially attenuate the association signal between AAM, ANM, and sepsis, leading to conservative results.

## Conclusion

To summarize, our study offers compelling evidence indicating a link between early AAM and a heightened risk of sepsis, while no causative association was established between ANM and sepsis. These findings contribute to our comprehension of sepsis pathogenesis and suggest that AAM might merit consideration as a potential risk factor for sepsis.

## Supporting information

S1 File (DOCX)

S1 Table Description of data sources about the MR analyses. (DOCX)

S2 Table Detailed information for the genetic variants associated with AAM. (DOCX)

S3 Table Detailed information for the genetic variants associated with ANM. (DOCX)
