# Supplementary material for: The causal effects of age at menarche and age at menopause on sepsis: A two-sample Mendelian randomization analysis
Source: PLoS One. 2024 Feb 7;19(2):e0293540. doi: 10.1371/journal.pone.0293540 (PMC10849219; doi:10.1371/journal.pone.0293540)
Supplement: S1 File — (DOCX) [file pone.0293540.s001.docx]

AAM and sepsis R code：

> library(VariantAnnotation)

载入需要的程辑包：BiocGenerics

载入程辑包：‘BiocGenerics’

The following objects are masked from ‘package:stats’:

IQR, mad, sd, var, xtabs

The following objects are masked from ‘package:base’:

anyDuplicated, aperm, append, as.data.frame,

basename, cbind, colnames, dirname, do.call,

duplicated, eval, evalq, Filter, Find, get, grep,

grepl, intersect, is.unsorted, lapply, Map, mapply,

match, mget, order, paste, pmax, pmax.int, pmin,

pmin.int, Position, rank, rbind, Reduce, rownames,

sapply, setdiff, sort, table, tapply, union, unique,

unsplit, which.max, which.min

载入需要的程辑包：MatrixGenerics

载入需要的程辑包：matrixStats

载入程辑包：‘MatrixGenerics’

The following objects are masked from ‘package:matrixStats’:

colAlls, colAnyNAs, colAnys, colAvgsPerRowSet,

colCollapse, colCounts, colCummaxs, colCummins,

colCumprods, colCumsums, colDiffs, colIQRDiffs,

colIQRs, colLogSumExps, colMadDiffs, colMads,

colMaxs, colMeans2, colMedians, colMins,

colOrderStats, colProds, colQuantiles, colRanges,

colRanks, colSdDiffs, colSds, colSums2, colTabulates,

colVarDiffs, colVars, colWeightedMads,

colWeightedMeans, colWeightedMedians, colWeightedSds,

colWeightedVars, rowAlls, rowAnyNAs, rowAnys,

rowAvgsPerColSet, rowCollapse, rowCounts, rowCummaxs,

rowCummins, rowCumprods, rowCumsums, rowDiffs,

rowIQRDiffs, rowIQRs, rowLogSumExps, rowMadDiffs,

rowMads, rowMaxs, rowMeans2, rowMedians, rowMins,

rowOrderStats, rowProds, rowQuantiles, rowRanges,

rowRanks, rowSdDiffs, rowSds, rowSums2, rowTabulates,

rowVarDiffs, rowVars, rowWeightedMads,

rowWeightedMeans, rowWeightedMedians, rowWeightedSds,

rowWeightedVars

载入需要的程辑包：GenomeInfoDb

载入需要的程辑包：S4Vectors

载入需要的程辑包：stats4

载入程辑包：‘S4Vectors’

The following object is masked from ‘package:utils’:

findMatches

The following objects are masked from ‘package:base’:

expand.grid, I, unname

载入需要的程辑包：IRanges

载入程辑包：‘IRanges’

The following object is masked from ‘package:grDevices’:

windows

载入需要的程辑包：GenomicRanges

载入需要的程辑包：SummarizedExperiment

载入需要的程辑包：Biobase

Welcome to Bioconductor

Vignettes contain introductory material; view with

'browseVignettes()'. To cite Bioconductor, see

'citation("Biobase")', and for packages

'citation("pkgname")'.

载入程辑包：‘Biobase’

The following object is masked from ‘package:MatrixGenerics’:

rowMedians

The following objects are masked from ‘package:matrixStats’:

anyMissing, rowMedians

载入需要的程辑包：Rsamtools

载入需要的程辑包：Biostrings

载入需要的程辑包：XVector

载入程辑包：‘Biostrings’

The following object is masked from ‘package:base’:

strsplit

载入程辑包：‘VariantAnnotation’

The following object is masked from ‘package:base’:

tabulate

> library(gwasglue)

载入需要的程辑包：gwasvcf

载入需要的程辑包：ieugwasr

API: public: http://gwas-api.mrcieu.ac.uk/

载入程辑包：‘ieugwasr’

The following object is masked from ‘package:IRanges’:

cor

The following object is masked from ‘package:S4Vectors’:

cor

> setwd("D:/桌面/Age at menarche")

> bim_VCF=readVcf("ieu-a-1095.vcf.gz")

> bmi=gwasvcf_to_TwoSampleMR(vcf=bim_VCF,type="exposure")

> bmi_head =head(bmi)

> View(bmi_head)

> write.csv(bmi,file="exposure.csv")

> a<-read.table("exposure.csv",header=T,sep = ",") #读入数据

> View(a)

> b<-subset(a,pval.exposure<5e-08) #看表格是是哪一种表示的p，保持一致

> View(b)

> #已完成相关性设置 （subset函数）

> write.csv(b,file="exposure.csv")

> #读取exposure数据，一定要将相关性设置好的暴露数据放入到 TwoSampleMR package包的文件夹中

> bmi<-system.file("exposure.csv",package="TwoSampleMR")

> #调整clump的参数 （进行独立性设置）

> bmi_exp_dat<-read_exposure_data(filename=bmi, sep=",",snp_col="SNP",beta_col="beta",se_col="se",effect_allele_col="effect_allele",other_allele_col="other_allele",eaf_col="eaf")

Error in read_exposure_data(filename = bmi, sep = ",", snp_col = "SNP", :

could not find function "read_exposure_data"

> bmi_exp_dat_clumped<-read_exposure_data(filename=bmi, sep=",",snp_col="SNP",beta_col="beta",se_col="se",effect_allele_col="effect_allele",other_allele_col="other_allele",eaf_col="eaf",clump=TRUE)

Error in read_exposure_data(filename = bmi, sep = ",", snp_col = "SNP", :

could not find function "read_exposure_data"

> library(TwoSampleMR)

TwoSampleMR version 0.5.6

[>] New: Option to use non-European LD reference panels for clumping etc

[>] Some studies temporarily quarantined to verify effect allele

[>] See news(package='TwoSampleMR') and https://gwas.mrcieu.ac.uk for further details

Warning:

You are running an old version of the TwoSampleMR package.

This version: 0.5.6

Latest version: 0.5.7

Please consider updating using remotes::install_github('MRCIEU/TwoSampleMR')

载入程辑包：‘TwoSampleMR’

The following object is masked from ‘package:ieugwasr’:

ld_matrix

The following object is masked from ‘package:SummarizedExperiment’:

trim

The following object is masked from ‘package:GenomicRanges’:

trim

The following object is masked from ‘package:IRanges’:

trim

> bmi<-system.file("exposure.csv",package="TwoSampleMR")

> #调整clump的参数 （进行独立性设置）

> bmi_exp_dat<-read_exposure_data(filename=bmi, sep=",",snp_col="SNP",beta_col="beta",se_col="se",effect_allele_col="effect_allele",other_allele_col="other_allele",eaf_col="eaf")

No phenotype name specified, defaulting to 'exposure'.

Inferring p-values

> bmi_exp_dat_clumped<-read_exposure_data(filename=bmi, sep=",",snp_col="SNP",beta_col="beta",se_col="se",effect_allele_col="effect_allele",other_allele_col="other_allele",eaf_col="eaf",clump=TRUE)

No phenotype name specified, defaulting to 'exposure'.

Inferring p-values

Please look at vignettes for options on running this locally if you need to run many instances of this command.

Clumping 1zmQyh, 2460 variants, using EUR population reference

Server code: 502; Server is possibly experiencing traffic, trying again...

Server code: 502; Server is possibly experiencing traffic, trying again...

Server code: 502; Server is possibly experiencing traffic, trying again...

Retry succeeded!

Removing 2391 of 2460 variants due to LD with other variants or absence from LD reference panel

> #设置工作环境

> #第二步 OUTCOME DATA

> #结局数据选择的注意要点：是否和暴露数据有重叠sample overlapping？结局数据中的SNP量是否足够多？ 一般要500万左右起步，要是太少的话会丢失太多暴露筛选出来的SNP。

> #导入OUTCOME 数据

> #R program 读取Outcome full GWAS data

> c<-read.table("sepsis.csv",header=T,sep = ",")

> d<-merge(bmi_exp_dat_clumped,c,by.x="SNP",by.y="SNP")#对应两个文件的SNP名字

> View(d)

> write.csv(d,file="outcome.csv")

> outcome_dat <-read_outcome_data(

+ snps=bmi_exp_dat_clumped$SNP, #结局的snps与暴露的snp那栏对应#

+ filename="outcome.csv",

+ sep=",",

+ snp_col="SNP",

+ beta_col="beta",

+ se_col="se",

+ effect_allele_col="effect_allele",

+ other_allele_col="other_allele", #eaf_col数据没有就不分析#

+ pval_col="p")

No phenotype name specified, defaulting to 'outcome'.

> dat<-harmonise_data(exposure_dat = bmi_exp_dat_clumped,outcome_dat=outcome_dat)

Harmonising exposure (1zmQyh) and outcome (PlGJwY)

Removing the following SNPs for being palindromic with intermediate allele frequencies:

rs1518080, rs1874984, rs4242496, rs4801589, rs9373571, rs9939609

> write.csv(dat,file="harmonise_data.csv")

> mr(dat)

Analysing '1zmQyh' on 'PlGJwY'

id.exposure id.outcome outcome exposure

1 1zmQyh PlGJwY outcome exposure

2 1zmQyh PlGJwY outcome exposure

3 1zmQyh PlGJwY outcome exposure

4 1zmQyh PlGJwY outcome exposure

5 1zmQyh PlGJwY outcome exposure

method nsnp b se

1 MR Egger 61 -0.07391941 0.17986343

2 Weighted median 61 -0.13212862 0.06726670

3 Inverse variance weighted 61 -0.13906480 0.04754581

4 Simple mode 61 -0.11695242 0.14607667

5 Weighted mode 61 -0.13877860 0.11636255

pval

1 0.682579623

2 0.049501089

3 0.003446119

4 0.426508539

5 0.237705759

> generate_odds_ratios(mr_res=mr(dat))

Analysing '1zmQyh' on 'PlGJwY'

id.exposure id.outcome outcome exposure

1 1zmQyh PlGJwY outcome exposure

2 1zmQyh PlGJwY outcome exposure

3 1zmQyh PlGJwY outcome exposure

4 1zmQyh PlGJwY outcome exposure

5 1zmQyh PlGJwY outcome exposure

method nsnp b se

1 MR Egger 61 -0.07391941 0.17986343

2 Weighted median 61 -0.13212862 0.06479334

3 Inverse variance weighted 61 -0.13906480 0.04754581

4 Simple mode 61 -0.11695242 0.15067651

5 Weighted mode 61 -0.13877860 0.10732251

pval lo_ci up_ci or or_lci95

1 0.682579623 -0.4264517 0.278612908 0.9287465 0.6528214

2 0.041426942 -0.2591236 -0.005133665 0.8762283 0.7717277

3 0.003446119 -0.2322546 -0.045875020 0.8701716 0.7927443

4 0.440690418 -0.4122784 0.178373528 0.8896275 0.6621399

5 0.200933746 -0.3491307 0.071573521 0.8704207 0.7053009

or_uci95

1 1.3212958

2 0.9948795

3 0.9551613

4 1.1952717

5 1.0741971

> #使用固定方法

> mr(dat,method_list=c("mr_ivw","mr_weighted_median","mr_egger_regression","mr_weighted_mode","mr_simple_mode"))

Analysing '1zmQyh' on 'PlGJwY'

id.exposure id.outcome outcome exposure

1 1zmQyh PlGJwY outcome exposure

2 1zmQyh PlGJwY outcome exposure

3 1zmQyh PlGJwY outcome exposure

4 1zmQyh PlGJwY outcome exposure

5 1zmQyh PlGJwY outcome exposure

method nsnp b se

1 Inverse variance weighted 61 -0.13906480 0.04754581

2 Weighted median 61 -0.13212862 0.06767342

3 MR Egger 61 -0.07391941 0.17986343

4 Weighted mode 61 -0.13877860 0.11054372

5 Simple mode 61 -0.11695242 0.14737232

pval

1 0.003446119

2 0.050885406

3 0.682579623

4 0.214194331

5 0.430564536

> mr_scatter_plot(mr_results=mr(dat,method_list=c("mr_ivw","mr_weighted_median","mr_egger_regression","mr_weighted_mode","mr_simple_mode")),dat)

Analysing '1zmQyh' on 'PlGJwY'

$`1zmQyh.PlGJwY`

attr(,"split_type")

[1] "data.frame"

attr(,"split_labels")

id.exposure id.outcome

1 1zmQyh PlGJwY

> mr_heterogeneity(dat)#异质性一般看IVW的p值

id.exposure id.outcome outcome exposure

1 1zmQyh PlGJwY outcome exposure

2 1zmQyh PlGJwY outcome exposure

method Q Q_df Q_pval

1 MR Egger 73.39113 59 0.09844035

2 Inverse variance weighted 73.56677 60 0.11206472

> #异质性可视化

> mr_funnel_plot(singlesnp_results=mr_singlesnp(dat))

$`1zmQyh.PlGJwY`

attr(,"split_type")

[1] "data.frame"

attr(,"split_labels")

id.exposure id.outcome

1 1zmQyh PlGJwY

> #第六步 多效性检测 mr_pleiotropy_test()

> #1.何为多效性；一个SNP不仅与研究的暴露的相关，还与其他的表型相关，且这个表型还恰好影响结局的发生。

> #2.结果解读；3.多效性的影响。 出现多效性表明结果不靠谱，就不用往下做了。#p必须大于0.05，否则IVW结果不可靠。

> mr_pleiotropy_test(dat)

id.exposure id.outcome outcome exposure egger_intercept

1 1zmQyh PlGJwY outcome exposure -0.003121001

se pval

1 0.008305889 0.7084448

> #第七步 留一分析 leave one out analysis

> mr_leaveoneout_plot(leaveoneout_results=mr_leaveoneout(dat))

$`1zmQyh.PlGJwY`

attr(,"split_type")

[1] "data.frame"

attr(,"split_labels")

id.exposure id.outcome

1 1zmQyh PlGJwY

Warning messages:

1: Removed 1 rows containing missing values (`geom_errorbarh()`).

2: Removed 1 rows containing missing values (`geom_point()`).

ANM and sepsis R code：

version 4.3.0 (2023-04-21 ucrt) -- "Already Tomorrow"

Copyright (C) 2023 The R Foundation for Statistical Computing

Platform: x86_64-w64-mingw32/x64 (64-bit)

R is free software and comes with ABSOLUTELY NO WARRANTY.

You are welcome to redistribute it under certain conditions.

Type 'license()' or 'licence()' for distribution details.

R is a collaborative project with many contributors.

Type 'contributors()' for more information and

'citation()' on how to cite R or R packages in publications.

Type 'demo()' for some demos, 'help()' for on-line help, or

'help.start()' for an HTML browser interface to help.

Type 'q()' to quit R.

[Workspace loaded from ~/.RData]

> a<-read.table("exposure.csv",header=T,sep = ",") #读入数据

Error in file(file, "rt") : cannot open the connection

In addition: Warning message:

In file(file, "rt") :

cannot open file 'exposure.csv': No such file or directory

> setwd("D:/桌面/ANM")

> b<-read.table("exposure.csv",header=T,sep = ",") #读入数据

> #独立性设置 （TwoSampleMR中的clump函数）

> library(TwoSampleMR)

TwoSampleMR version 0.5.6

[>] New: Option to use non-European LD reference panels for clumping etc

[>] Some studies temporarily quarantined to verify effect allele

[>] See news(package='TwoSampleMR') and https://gwas.mrcieu.ac.uk for further details

Warning:

You are running an old version of the TwoSampleMR package.

This version: 0.5.6

Latest version: 0.5.7

Please consider updating using remotes::install_github('MRCIEU/TwoSampleMR')

> #读取exposure数据，一定要将相关性设置好的暴露数据放入到 TwoSampleMR package包的文件夹中

> bmi<-system.file("exposure.csv",package="TwoSampleMR")

> bmi_exp_dat_clumped<-read_exposure_data(filename=bmi, sep=",",snp_col="SNP",beta_col="beta",se_col="se",effect_allele_col="effect_allele",other_allele_col="other_allele",eaf_col="eaf",clump=TRUE)

No phenotype name specified, defaulting to 'exposure'.

Inferring p-values

API: public: http://gwas-api.mrcieu.ac.uk/

Please look at vignettes for options on running this locally if you need to run many instances of this command.

Clumping jnV9tm, 28525 variants, using EUR population reference

Server code: 502; Server is possibly experiencing traffic, trying again...

Retry succeeded!

Removing 28312 of 28525 variants due to LD with other variants or absence from LD reference panel

Warning message:

In format_data(as.data.frame(exposure_dat), type = "exposure", snps = NULL, :

The following columns are not present but are helpful for harmonisation

eaf

> #设置工作环境

> #第二步 OUTCOME DATA

> #结局数据选择的注意要点：是否和暴露数据有重叠sample overlapping？结局数据中的SNP量是否足够多？ 一般要500万左右起步，要是太少的话会丢失太多暴露筛选出来的SNP。

> #导入OUTCOME 数据

> #R program 读取Outcome full GWAS data

> c<-read.table("sepsis.csv",header=T,sep = ",")

> #Merge函数，提取OUTCOME数据（将暴露和结局数据取交集）

> d<-merge(bmi_exp_dat_clumped,c,by.x="SNP",by.y="SNP")#对应两个文件的SNP名字

> outcome<-extract_outcome_data(snps=bmi_exp_dat_clumped$SNP,outcomes="ieu-b-4980")

Extracting data for 213 SNP(s) from 1 GWAS(s)

Finding proxies for 31 SNPs in outcome ieu-b-4980

Extracting data for 31 SNP(s) from 1 GWAS(s)

> write.csv(outcome,file="outcome_proxy.csv")

> #第三步 Harmonize data

> #协同：作用是为了将Exposure-SNP及Outcome-SNP等位基因方向协同；根据EAF大小，剔除不能判断方向的palindromic SNP (回文序列); 剔除incompatible SNP (A/G vs. A/C)(不兼容序列)。

> dat<-harmonise_data(exposure_dat = bmi_exp_dat_clumped,outcome_dat=outcome)

Harmonising exposure (jnV9tm) and Sepsis || id:ieu-b-4980 (ieu-b-4980)

Removing the following SNPs for being palindromic with intermediate allele frequencies:

rs10823203, rs10854167, rs11071756, rs112097885, rs11719186, rs117431243, rs11767307, rs12605881, rs131658, rs1476164, rs158673, rs200293726, rs2023776, rs2052160, rs2758605, rs28652789, rs2885255, rs2958663, rs35067339, rs35536133, rs3736830, rs3750243, rs3796624, rs3803756, rs426564, rs4668354, rs4731541, rs55863203, rs62244773, rs6430545, rs67893326, rs728900, rs73046547, rs7515939, rs7567184, rs7609266, rs8100087, rs907694, rs9264669, rs9796

> write.csv(dat,file="harmonise_data.csv")

> dat<-subset(dat,mr_keep==T)

> #第四步 Perform MR MR分析

> #MR Estimation

> #1.run "mr()"function in the TwoSampleMR

> #2.如何利用mr()函数一次性跑出自己想要的数据

> #3.数据如何解读#IVW的P值＜0.05，其他的大于0.05也可以当作阳性结果，但其他方法的beta值与IVW方向必须一致，当然最好是所有方向的P值都小于0.05。

> #4.如何进行对数转化

> #5.如何可视化

> mr(dat)

Analysing 'jnV9tm' on 'ieu-b-4980'

id.exposure id.outcome outcome exposure

1 jnV9tm ieu-b-4980 Sepsis || id:ieu-b-4980 exposure

2 jnV9tm ieu-b-4980 Sepsis || id:ieu-b-4980 exposure

3 jnV9tm ieu-b-4980 Sepsis || id:ieu-b-4980 exposure

4 jnV9tm ieu-b-4980 Sepsis || id:ieu-b-4980 exposure

5 jnV9tm ieu-b-4980 Sepsis || id:ieu-b-4980 exposure

method nsnp b se pval

1 MR Egger 166 -0.006230130 0.015689087 0.6918103

2 Weighted median 166 0.007144143 0.014491577 0.6220226

3 Inverse variance weighted 166 -0.012829773 0.008451402 0.1289982

4 Simple mode 166 0.008718781 0.030346984 0.7742408

5 Weighted mode 166 0.004453869 0.016197531 0.7836817

> generate_odds_ratios(mr_res=mr(dat))

Analysing 'jnV9tm' on 'ieu-b-4980'

id.exposure id.outcome outcome exposure

1 jnV9tm ieu-b-4980 Sepsis || id:ieu-b-4980 exposure

2 jnV9tm ieu-b-4980 Sepsis || id:ieu-b-4980 exposure

3 jnV9tm ieu-b-4980 Sepsis || id:ieu-b-4980 exposure

4 jnV9tm ieu-b-4980 Sepsis || id:ieu-b-4980 exposure

5 jnV9tm ieu-b-4980 Sepsis || id:ieu-b-4980 exposure

method nsnp b se pval

1 MR Egger 166 -0.006230130 0.015689087 0.6918103

2 Weighted median 166 0.007144143 0.014379615 0.6193129

3 Inverse variance weighted 166 -0.012829773 0.008451402 0.1289982

4 Simple mode 166 0.008718781 0.030894321 0.7781338

5 Weighted mode 166 0.004453869 0.015836274 0.7788751

lo_ci up_ci or or_lci95 or_uci95

1 -0.03698074 0.024520481 0.9937892 0.9636947 1.024824

2 -0.02103990 0.035328188 1.0071697 0.9791799 1.035960

3 -0.02939452 0.003734976 0.9872522 0.9710333 1.003742

4 -0.05183409 0.069271651 1.0087569 0.9494864 1.071727

5 -0.02658523 0.035492967 1.0044638 0.9737650 1.036130

> mr_heterogeneity(dat)#异质性一般看IVW的p值

id.exposure id.outcome outcome exposure

1 jnV9tm ieu-b-4980 Sepsis || id:ieu-b-4980 exposure

2 jnV9tm ieu-b-4980 Sepsis || id:ieu-b-4980 exposure

method Q Q_df Q_pval

1 MR Egger 161.7065 164 0.5360036

2 Inverse variance weighted 161.9557 165 0.5524511

> #看异质性来自哪

> #run_mr_presso(dat,NbDistribution=10000)

>

>

> #异质性可视化

> mr_funnel_plot(singlesnp_results=mr_singlesnp(dat))

$`jnV9tm.ieu-b-4980`

attr(,"split_type")

[1] "data.frame"

attr(,"split_labels")

id.exposure id.outcome

1 jnV9tm ieu-b-4980

>

>

>

> #第六步 多效性检测 mr_pleiotropy_test()

> #1.何为多效性；一个SNP不仅与研究的暴露的相关，还与其他的表型相关，且这个表型还恰好影响结局的发生。

> #2.结果解读；3.多效性的影响。 出现多效性表明结果不靠谱，就不用往下做了。#p必须大于0.05，否则IVW结果不可靠。

> mr_pleiotropy_test(dat)

id.exposure id.outcome outcome exposure

1 jnV9tm ieu-b-4980 Sepsis || id:ieu-b-4980 exposure

egger_intercept se pval

1 -0.001181903 0.002367196 0.6182485

>

>

> #第七步 留一分析 leave one out analysis

> mr_leaveoneout_plot(leaveoneout_results=mr_leaveoneout(dat))

$`jnV9tm.ieu-b-4980`

attr(,"split_type")

[1] "data.frame"

attr(,"split_labels")

id.exposure id.outcome

1 jnV9tm ieu-b-4980

Warning messages:

1: Removed 1 rows containing missing values (`geom_errorbarh()`).

2: Removed 1 rows containing missing values (`geom_point()`).

> mr_scatter_plot(mr_results=mr(dat,method_list=c("mr_ivw","mr_weighted_median","mr_egger_regression","mr_weighted_mode","mr_simple_mode")),dat)

Analysing 'jnV9tm' on 'ieu-b-4980'

$`jnV9tm.ieu-b-4980`

attr(,"split_type")

[1] "data.frame"

attr(,"split_labels")

id.exposure id.outcome

1 jnV9tm ieu-b-4980
