# Supplementary material for: The causal effects of age at menarche and age at menopause on sepsis: A two-sample Mendelian randomization analysis
Source: PLoS One. 2024 Feb 7;19(2):e0293540. doi: 10.1371/journal.pone.0293540 (PMC10849219; doi:10.1371/journal.pone.0293540)
Supplement: S1 Table — (DOCX) [file pone.0293540.s002.docx]

S1 Table. Description of data sources about the MR analyses.

| Trait | Consortium | Sample size | Population |
| --- | --- | --- | --- |
| AAM | ReproGen(2014) | 182,416 females | European |
| ANM | ReproGen(2021) | 201,323 females | European |
| Sepsis | UK Biobank(2021) | 486,484 Males and Females | European |
