# Supplementary material for: The causal effects of age at menarche and age at menopause on sepsis: A two-sample Mendelian randomization analysis
Source: PLoS One. 2024 Feb 7;19(2):e0293540. doi: 10.1371/journal.pone.0293540 (PMC10849219; doi:10.1371/journal.pone.0293540)
Supplement: S2 Table — (DOCX) [file pone.0293540.s003.docx]

S2 Table. Detailed information for the genetic variants associated with AAM.

| SNP | Effect allele | Other allele | Eaf | Beta | SE | pval | *F*-statistic |
| --- | --- | --- | --- | --- | --- | --- | --- |
| rs10144321 | G | A | 0.22 | -0.042 | 0.007 | 2.20E-10 | 110.501 |
| rs10483727 | C | T | 0.64 | -0.037 | 0.006 | 1.60E-10 | 115.146 |
| rs1079866 | G | C | 0.12 | 0.072 | 0.008 | 1.50E-17 | 199.937 |
| rs10840031 | A | G | 0.24 | 0.038 | 0.006 | 3.00E-09 | 96.141 |
| rs10938397 | G | A | 0.43 | -0.038 | 0.006 | 1.40E-10 | 129.213 |
| rs11022756 | C | A | 0.74 | -0.048 | 0.006 | 1.30E-13 | 161.868 |
| rs11715566 | T | C | 0.51 | 0.052 | 0.006 | 2.60E-20 | 246.859 |
| rs11767400 | A | C | 0.31 | 0.035 | 0.006 | 2.70E-08 | 95.645 |
| rs12003641 | T | C | 0.07 | 0.082 | 0.011 | 7.90E-15 | 159.837 |
| rs12148769 | A | G | 0.12 | -0.055 | 0.010 | 1.70E-08 | 116.615 |
| rs12291726 | G | A | 0.11 | 0.057 | 0.008 | 7.90E-12 | 116.117 |
| rs12598642 | G | A | 0.44 | 0.044 | 0.006 | 9.50E-15 | 174.200 |
| rs12915845 | T | C | 0.42 | -0.035 | 0.006 | 1.00E-09 | 108.933 |
| rs13179411 | T | G | 0.16 | 0.06 | 0.008 | 1.60E-14 | 176.689 |
| rs13215865 | T | C | 0.15 | -0.042 | 0.007 | 1.90E-08 | 82.090 |
| rs1482853 | A | C | 0.39 | -0.038 | 0.006 | 3.20E-10 | 125.415 |
| rs1516883 | A | G | 0.3 | -0.091 | 0.002 | 1.00E-200 | 636.654 |
| rs1659127 | A | G | 0.3 | 0.044 | 0.006 | 5.00E-12 | 148.445 |
| rs16938437 | T | C | 0.05 | -0.067 | 0.010 | 9.50E-11 | 77.825 |
| rs17351680 | G | C | 0.13 | 0.044 | 0.008 | 5.80E-09 | 79.918 |
| rs2179786 | T | G | 0.32 | -0.039 | 0.006 | 9.20E-12 | 120.827 |
| rs2184968 | C | T | 0.41 | -0.036 | 0.006 | 2.20E-10 | 114.446 |
| rs2303100 | T | C | 0.55 | 0.038 | 0.006 | 2.30E-11 | 130.479 |
| rs2344508 | A | G | 0.56 | 0.034 | 0.006 | 3.30E-09 | 103.976 |
| rs2617056 | T | A | 0.39 | -0.036 | 0.006 | 1.30E-09 | 112.553 |
| rs2684838 | G | T | 0.57 | 0.045 | 0.006 | 7.20E-15 | 181.254 |
| rs2687729 | G | A | 0.23 | 0.044 | 0.007 | 2.20E-11 | 125.173 |
| rs2836950 | G | C | 0.32 | -0.035 | 0.006 | 1.70E-08 | 97.300 |
| rs2947411 | G | A | 0.87 | -0.052 | 0.008 | 1.10E-11 | 111.641 |
| rs3115627 | G | A | 0.4 | 0.038 | 0.006 | 3.90E-09 | 126.522 |
| rs3733632 | G | A | 0.23 | 0.049 | 0.008 | 4.60E-10 | 155.263 |
| rs3743266 | C | T | 0.33 | -0.045 | 0.006 | 5.20E-13 | 163.490 |
| rs3870341 | G | A | 0.69 | -0.043 | 0.006 | 2.60E-11 | 144.404 |
| rs4369815 | G | T | 0.05 | -0.08 | 0.012 | 1.70E-11 | 110.975 |
| rs466639 | C | T | 0.87 | 0.075 | 0.009 | 7.00E-18 | 232.395 |
| rs4735766 | T | G | 0.35 | -0.047 | 0.006 | 4.70E-13 | 183.528 |
| rs4840086 | G | A | 0.48 | -0.036 | 0.006 | 1.50E-10 | 118.092 |
| rs618678 | T | C | 0.33 | -0.034 | 0.006 | 3.20E-08 | 93.295 |
| rs633715 | C | T | 0.2 | -0.051 | 0.007 | 2.00E-12 | 151.953 |
| rs6694738 | A | C | 0.87 | -0.052 | 0.008 | 1.10E-11 | 111.641 |
| rs6747380 | A | G | 0.17 | 0.065 | 0.008 | 2.10E-17 | 217.751 |
| rs6758290 | C | T | 0.51 | -0.04 | 0.006 | 2.50E-10 | 145.990 |
| rs6770162 | A | G | 0.44 | 0.036 | 0.006 | 3.40E-10 | 116.577 |
| rs6933660 | A | C | 0.3 | -0.036 | 0.006 | 1.30E-08 | 99.346 |
| rs7103411 | T | C | 0.78 | -0.043 | 0.007 | 1.10E-09 | 115.829 |
| rs7119712 | A | G | 0.19 | -0.041 | 0.006 | 2.00E-10 | 94.432 |
| rs740077 | C | A | 0.21 | -0.046 | 0.007 | 6.60E-11 | 128.161 |
| rs7642134 | G | A | 0.56 | 0.038 | 0.006 | 1.30E-10 | 129.899 |
| rs7759932 | C | T | 0.46 | -0.08 | 0.002 | 1.00E-200 | 581.839 |
| rs7853970 | C | T | 0.57 | -0.037 | 0.006 | 3.40E-09 | 122.497 |
| rs7944630 | A | G | 0.57 | 0.047 | 0.006 | 2.30E-16 | 197.741 |
| rs8012970 | C | T | 0.68 | -0.034 | 0.006 | 5.00E-08 | 91.817 |
| rs852069 | G | A | 0.62 | 0.036 | 0.006 | 1.10E-09 | 111.464 |
| rs888345 | A | G | 0.82 | -0.044 | 0.007 | 1.80E-09 | 104.311 |
| rs895526 | C | T | 0.8 | 0.044 | 0.008 | 5.70E-09 | 113.079 |
| rs913588 | A | G | 0.55 | -0.034 | 0.006 | 2.20E-09 | 104.441 |
| rs9555810 | G | C | 0.26 | 0.047 | 0.006 | 4.50E-13 | 155.188 |
| rs9565073 | C | T | 0.52 | 0.034 | 0.006 | 7.40E-09 | 105.327 |
| rs9635759 | A | G | 0.33 | 0.058 | 0.006 | 7.70E-20 | 271.756 |
| rs9939609 | A | T | 0.45 | -0.042 | 0.006 | 2.10E-13 | 159.419 |
| rs9997604 | C | A | 0.7 | 0.039 | 0.007 | 2.10E-09 | 116.604 |
