# Supplementary material for: The causal effects of age at menarche and age at menopause on sepsis: A two-sample Mendelian randomization analysis
Source: PLoS One. 2024 Feb 7;19(2):e0293540. doi: 10.1371/journal.pone.0293540 (PMC10849219; doi:10.1371/journal.pone.0293540)
Supplement: S3 Table — (DOCX) [file pone.0293540.s004.docx]

**S3 Table.** Detailed information for the genetic variants associated with ANM.

| **SNP** | **Effect allele** | **Other allele** | **Eaf** | **Beta** | **SE** | **pval** | **F-statistic** |
| --- | --- | --- | --- | --- | --- | --- | --- |
| rs200448 | T | C | 0.5619 | -0.0993 | 0.0122 | 4.01E-16 | 982.1172809 |
| rs201387068 | T | A | 0.1532 | 0.209 | 0.02 | 1.55E-25 | 2307.817283 |
| rs4970634 | G | A | 0.6783 | -0.2142 | 0.0129 | 9.44E-62 | 4113.53561 |
| rs12046563 | A | G | 0.2391 | -0.1004 | 0.0142 | 1.72E-12 | 741.1216329 |
| rs55707872 | A | C | 0.3129 | 0.1531 | 0.0132 | 2.59E-31 | 2049.718933 |
| rs12133213 | G | A | 0.5343 | -0.0804 | 0.0129 | 5.30E-10 | 649.7135213 |
| rs72708144 | T | C | 0.0404 | 0.2816 | 0.0319 | 1.11E-18 | 1245.473434 |
| rs184655369 | G | T | 0.0934 | 0.1647 | 0.0249 | 3.89E-11 | 929.1130455 |
| rs11582336 | A | G | 0.2049 | -0.1056 | 0.015 | 2.03E-12 | 734.1613081 |
| rs1044595 | C | T | 0.5979 | 0.1323 | 0.0124 | 1.27E-26 | 1708.724061 |
| rs7414807 | G | A | 0.6326 | 0.0879 | 0.0127 | 3.99E-12 | 725.6509053 |
| rs1635506 | T | C | 0.4791 | 0.1495 | 0.0123 | 2.86E-34 | 2271.19294 |
| rs7539755 | C | T | 0.4234 | -0.1012 | 0.0122 | 1.36E-16 | 1011.772205 |
| rs72764660 | C | T | 0.0649 | 0.1386 | 0.0251 | 3.35E-08 | 470.5026614 |
| rs115880416 | G | A | 0.0409 | -0.2101 | 0.0309 | 1.05E-11 | 699.6244988 |
| rs704795 | G | A | 0.394 | -0.1986 | 0.0123 | 3.35E-58 | 3864.596907 |
| rs12053063 | A | G | 0.7212 | 0.1032 | 0.0135 | 2.37E-14 | 865.9463597 |
| rs17425341 | T | G | 0.3433 | -0.0847 | 0.0127 | 2.35E-11 | 653.3318282 |
| rs76928871 | A | G | 0.1896 | 0.1976 | 0.0155 | 2.08E-37 | 2444.96865 |
| rs72886832 | T | C | 0.1342 | -0.098 | 0.0178 | 3.45E-08 | 450.3100692 |
| rs62156756 | A | G | 0.1037 | -0.2629 | 0.0198 | 2.18E-40 | 2620.284642 |
| rs6727266 | A | G | 0.5057 | 0.0792 | 0.0122 | 9.89E-11 | 633.311029 |
| rs72827480 | T | C | 0.4013 | 0.1223 | 0.0126 | 2.66E-22 | 1457.41438 |
| rs16830019 | G | T | 0.2626 | 0.1183 | 0.0139 | 2.11E-17 | 1097.101191 |
| rs2357064 | G | A | 0.8841 | -0.1125 | 0.0188 | 2.30E-09 | 523.5242934 |
| rs72934556 | T | G | 0.1258 | 0.1009 | 0.0183 | 3.70E-08 | 451.821026 |
| rs6736096 | T | C | 0.5235 | 0.0982 | 0.012 | 3.14E-16 | 973.231284 |
| rs62193239 | G | A | 0.3388 | -0.074 | 0.0129 | 1.04E-08 | 495.1372168 |
| rs654448 | C | T | 0.8235 | 0.148 | 0.0158 | 7.89E-21 | 1290.10478 |
| rs12487736 | C | T | 0.5795 | -0.0775 | 0.0123 | 3.51E-10 | 591.0375213 |
| rs9968117 | C | T | 0.1188 | 0.1057 | 0.0188 | 1.80E-08 | 472.0381269 |
| rs7610102 | G | A | 0.3948 | -0.0766 | 0.0126 | 1.26E-09 | 566.0725674 |
| rs4679244 | A | G | 0.4133 | -0.1089 | 0.0124 | 1.30E-18 | 1164.558482 |
| rs9879207 | T | C | 0.1741 | 0.0931 | 0.0165 | 1.54E-08 | 503.0701572 |
| rs6793835 | G | A | 0.2617 | -0.1407 | 0.0138 | 1.82E-24 | 1551.954324 |
| rs10154963 | C | T | 0.7202 | -0.0765 | 0.0136 | 1.81E-08 | 475.9576456 |
| rs344018 | G | A | 0.4156 | -0.0942 | 0.0122 | 1.32E-14 | 871.5306306 |
| rs10937153 | G | A | 0.2423 | 0.1438 | 0.014 | 1.25E-24 | 1540.272689 |
| rs10804920 | T | C | 0.5611 | -0.0842 | 0.0122 | 5.98E-12 | 705.4532975 |
| rs56401314 | C | T | 0.3293 | 0.083 | 0.0138 | 1.73E-09 | 614.4957484 |
| rs6824237 | T | C | 0.2494 | -0.0771 | 0.014 | 3.47E-08 | 449.055971 |
| rs76540949 | T | C | 0.4779 | -0.1407 | 0.0122 | 9.56E-31 | 2008.67503 |
| rs12651246 | G | A | 0.4875 | 0.2609 | 0.0121 | 2.19E-103 | 7088.665784 |
| rs3857226 | T | C | 0.4997 | -0.0796 | 0.012 | 3.94E-11 | 639.8278291 |
| rs6810489 | T | G | 0.4042 | -0.0902 | 0.0124 | 4.38E-13 | 792.0163781 |
| rs28785481 | G | A | 0.3443 | -0.1022 | 0.0127 | 9.03E-16 | 953.9289983 |
| rs112898082 | G | A | 0.2603 | 0.0963 | 0.0153 | 2.79E-10 | 721.5312703 |
| rs11940804 | T | C | 0.398 | 0.2066 | 0.0123 | 6.32E-63 | 4203.724719 |
| rs9990489 | C | T | 0.4101 | -0.0981 | 0.0126 | 8.51E-15 | 941.7858489 |
| rs274701 | C | A | 0.4079 | -0.144 | 0.0124 | 3.22E-31 | 2036.876722 |
| rs74896614 | G | A | 0.0567 | -0.1619 | 0.0266 | 1.25E-09 | 566.0634024 |
| rs62356073 | G | A | 0.5736 | -0.0697 | 0.0123 | 1.56E-08 | 479.5614457 |
| rs17206591 | A | C | 0.4366 | -0.0741 | 0.0122 | 1.35E-09 | 545.2940964 |
| rs7728833 | A | G | 0.5003 | -0.0793 | 0.0121 | 5.13E-11 | 634.9989112 |
| rs10070308 | C | T | 0.156 | -0.0994 | 0.0167 | 2.77E-09 | 525.1583771 |
| rs10051199 | G | T | 0.4755 | 0.0921 | 0.0121 | 3.35E-14 | 855.4128236 |
| rs888694 | A | C | 0.9138 | -0.1596 | 0.0216 | 1.32E-13 | 811.12827 |
| rs4042113 | C | T | 0.1761 | 0.1455 | 0.0159 | 6.73E-20 | 1244.385887 |
| rs2241584 | G | A | 0.382 | -0.1593 | 0.0125 | 3.24E-37 | 2441.391422 |
| rs11740768 | G | A | 0.4861 | 0.318 | 0.012 | 4.66E-154 | 10712.55512 |
| rs11752007 | T | C | 0.1258 | 0.1187 | 0.0189 | 3.64E-10 | 625.8351162 |
| rs4716056 | A | G | 0.3807 | 0.0759 | 0.0125 | 1.15E-09 | 548.3627128 |
| rs2844466 | T | C | 0.3579 | -0.2596 | 0.0129 | 7.68E-90 | 6435.134011 |
| rs186325882 | G | A | 0.3375 | -0.0946 | 0.0157 | 1.84E-09 | 808.9143134 |
| rs2817019 | A | G | 0.3664 | 0.0953 | 0.0129 | 1.51E-13 | 852.5320779 |
| rs515650 | G | T | 0.5544 | -0.0832 | 0.0123 | 1.64E-11 | 690.9108753 |
| rs113967617 | G | A | 0.1544 | 0.1215 | 0.0169 | 5.69E-13 | 779.0426365 |
| rs6569648 | C | T | 0.7642 | -0.1227 | 0.0142 | 6.95E-18 | 1098.302264 |
| rs9347077 | A | G | 0.1319 | -0.1066 | 0.0179 | 2.46E-09 | 525.2664684 |
| rs4709363 | G | A | 0.2779 | -0.0747 | 0.0136 | 3.82E-08 | 451.8766253 |
| rs10237045 | G | T | 0.3376 | 0.1008 | 0.0142 | 1.34E-12 | 919.0539248 |
| rs6949434 | C | T | 0.4784 | 0.0825 | 0.0121 | 9.30E-12 | 686.1727024 |
| rs62445870 | C | T | 0.0225 | 0.3161 | 0.0427 | 1.31E-13 | 888.7515417 |
| rs10255049 | G | A | 0.6879 | -0.1404 | 0.0131 | 1.02E-26 | 1718.557022 |
| rs2272343 | A | C | 0.2201 | -0.1065 | 0.0146 | 3.45E-13 | 786.9944445 |
| rs2392836 | G | A | 0.6274 | -0.1151 | 0.0125 | 3.98E-20 | 1254.744939 |
| rs112190116 | C | T | 0.0112 | 0.5911 | 0.0707 | 6.34E-17 | 1570.151294 |
| rs2061834 | T | G | 0.8047 | -0.1079 | 0.016 | 1.33E-11 | 739.418612 |
| rs6473979 | C | A | 0.9472 | -0.2128 | 0.027 | 3.59E-15 | 916.0296535 |
| rs3735828 | G | A | 0.6406 | -0.1151 | 0.0129 | 4.85E-19 | 1235.640503 |
| rs73264416 | A | G | 0.0771 | 0.1659 | 0.0228 | 3.25E-13 | 791.6354918 |
| rs1467044 | G | A | 0.5403 | -0.0695 | 0.0121 | 9.72E-09 | 484.2186013 |
| rs6470598 | C | T | 0.792 | -0.0853 | 0.0149 | 1.04E-08 | 483.7801264 |
| rs55873183 | A | G | 0.0664 | 0.1914 | 0.0264 | 4.25E-13 | 918.5629048 |
| rs4879656 | C | A | 0.3752 | -0.1329 | 0.0125 | 1.94E-26 | 1681.064054 |
| rs10818873 | T | C | 0.0647 | 0.2184 | 0.0248 | 1.24E-18 | 1168.94463 |
| rs113733516 | G | A | 0.5067 | -0.0703 | 0.0128 | 3.93E-08 | 498.615805 |
| rs10737018 | G | A | 0.8349 | -0.0922 | 0.0162 | 1.32E-08 | 472.9131979 |
| rs116963467 | A | G | 0.0564 | -0.2153 | 0.0269 | 1.27E-15 | 998.2106832 |
| rs10764106 | C | T | 0.4727 | -0.0775 | 0.0121 | 1.59E-10 | 604.6000188 |
| rs7087644 | A | G | 0.0424 | -0.2893 | 0.0306 | 3.23E-21 | 1377.614282 |
| rs7091889 | A | G | 0.214 | 0.1207 | 0.0148 | 2.82E-16 | 991.5249009 |
| rs61870304 | A | G | 0.111 | -0.1538 | 0.0223 | 5.26E-12 | 944.2536883 |
| rs6578283 | G | A | 0.7016 | -0.0777 | 0.0139 | 2.41E-08 | 510.2097525 |
| rs7928823 | A | G | 0.5715 | -0.0693 | 0.0124 | 2.21E-08 | 474.6520166 |
| rs201837298 | T | G | 0.8223 | 0.127 | 0.0191 | 3.01E-11 | 953.4461653 |
| rs11031005 | T | C | 0.1459 | 0.1999 | 0.0171 | 2.08E-31 | 2025.142255 |
| rs7125555 | C | T | 0.4749 | -0.1271 | 0.0121 | 1.35E-25 | 1635.187584 |
| rs7118226 | T | G | 0.791 | -0.0811 | 0.0148 | 4.55E-08 | 438.7620235 |
| rs12360650 | T | C | 0.1528 | -0.0954 | 0.0172 | 3.06E-08 | 475.4990722 |
| rs10899493 | T | C | 0.1664 | 0.1195 | 0.0162 | 1.86E-13 | 800.7369248 |
| rs7927748 | G | T | 0.5099 | -0.0751 | 0.0122 | 8.44E-10 | 569.1079132 |
| rs11611246 | G | T | 0.2137 | -0.098 | 0.0151 | 1.00E-10 | 651.8805214 |
| rs71579323 | A | G | 0.016 | 0.2926 | 0.0528 | 2.91E-08 | 544.1957774 |
| rs10743724 | T | C | 0.5197 | 0.0759 | 0.0121 | 3.48E-10 | 580.6557505 |
| rs12312134 | G | A | 0.311 | -0.0742 | 0.0131 | 1.32E-08 | 476.1375344 |
| rs2277339 | T | G | 0.1059 | -0.3696 | 0.0212 | 4.59E-68 | 5346.229441 |
| rs184540366 | G | T | 0.0228 | -0.4767 | 0.0509 | 8.16E-21 | 2059.433503 |
| rs75770066 | A | G | 0.0319 | 0.9606 | 0.0385 | 1.83E-137 | 12167.47606 |
| rs17180987 | G | A | 0.0183 | 0.3625 | 0.0485 | 8.28E-14 | 955.0373032 |
| rs1980236 | G | A | 0.4942 | -0.0671 | 0.0121 | 2.98E-08 | 454.1761633 |
| rs12371048 | C | T | 0.5512 | -0.0902 | 0.0122 | 1.59E-13 | 813.665549 |
| rs28416520 | G | A | 0.4622 | -0.168 | 0.0122 | 6.49E-43 | 2865.004237 |
| rs11571818 | T | C | 0.0092 | -0.5427 | 0.0657 | 1.47E-16 | 1086.802714 |
| rs7322160 | T | C | 0.3325 | 0.1864 | 0.0128 | 7.16E-48 | 3153.581501 |
| rs12868295 | C | T | 0.4079 | -0.0978 | 0.0124 | 2.84E-15 | 934.4513754 |
| rs1713426 | A | G | 0.3052 | -0.1545 | 0.0135 | 2.44E-30 | 2058.919531 |
| rs12879626 | T | G | 0.6124 | -0.0934 | 0.0126 | 1.04E-13 | 837.2093707 |
| rs61488898 | C | T | 0.0262 | -0.3556 | 0.0396 | 2.68E-19 | 1307.449681 |
| rs1969713 | T | C | 0.1044 | 0.1681 | 0.0201 | 5.34E-17 | 1069.473611 |
| rs112326803 | G | A | 0.0297 | 0.2538 | 0.0392 | 9.39E-11 | 750.2053274 |
| rs933941 | G | A | 0.1119 | 0.1349 | 0.0191 | 1.82E-12 | 730.8169505 |
| rs1982241 | T | C | 0.431 | 0.0733 | 0.0122 | 1.73E-09 | 531.9398918 |
| rs716886 | G | A | 0.3294 | 0.0837 | 0.0128 | 7.09E-11 | 625.0336272 |
| rs79719545 | G | A | 0.1115 | 0.1081 | 0.0195 | 3.02E-08 | 467.2071331 |
| rs12898357 | A | G | 0.3855 | -0.183 | 0.0125 | 1.47E-48 | 3245.738035 |
| rs11648292 | C | A | 0.695 | -0.0739 | 0.0134 | 3.18E-08 | 467.1961707 |
| rs17680522 | A | G | 0.2926 | 0.1254 | 0.0134 | 7.86E-21 | 1319.137044 |
| rs11075033 | C | T | 0.5968 | -0.2114 | 0.0123 | 9.23E-66 | 4425.077318 |
| rs559216182 | G | A | 0.1588 | -0.1433 | 0.0256 | 2.20E-08 | 1110.581428 |
| rs2173885 | G | A | 0.4608 | -0.1328 | 0.0129 | 9.01E-25 | 1779.919617 |
| rs59979850 | C | T | 0.481 | -0.0982 | 0.0126 | 7.94E-15 | 973.9810641 |
| rs11642909 | G | A | 0.6804 | 0.0776 | 0.0132 | 4.32E-09 | 528.6308122 |
| rs10521305 | T | C | 0.0597 | 0.2628 | 0.0289 | 9.62E-20 | 1573.22621 |
| rs8045027 | G | A | 0.5356 | -0.0728 | 0.0122 | 2.52E-09 | 532.1831701 |
| rs488327 | T | C | 0.3365 | 0.0763 | 0.0134 | 1.18E-08 | 524.7163591 |
| rs2108839 | T | G | 0.3097 | -0.136 | 0.0132 | 6.87E-25 | 1604.812076 |
| rs5030755 | A | G | 0.1174 | -0.1276 | 0.0191 | 2.42E-11 | 681.5854211 |
| rs34856659 | T | C | 0.2431 | 0.1677 | 0.0141 | 7.80E-33 | 2105.362183 |
| rs1565920 | G | A | 0.6811 | 0.1373 | 0.013 | 3.87E-26 | 1662.251273 |
| rs4796765 | A | G | 0.9391 | 0.1444 | 0.0261 | 3.01E-08 | 481.3041829 |
| rs799903 | G | A | 0.3345 | 0.176 | 0.0129 | 4.22E-42 | 2815.267606 |
| rs1991401 | A | G | 0.3306 | -0.1637 | 0.0138 | 1.72E-32 | 2416.500479 |
| rs34609096 | A | G | 0.5304 | -0.0681 | 0.0122 | 2.31E-08 | 466.1754346 |
| rs4800141 | G | A | 0.6516 | 0.0744 | 0.0127 | 4.24E-09 | 507.2441309 |
| rs10411808 | G | A | 0.1494 | -0.1051 | 0.0177 | 2.84E-09 | 566.7890554 |
| rs11670032 | T | C | 0.1443 | -0.1816 | 0.0174 | 1.56E-25 | 1653.065637 |
| rs73037453 | C | T | 0.189 | -0.1362 | 0.0155 | 1.38E-18 | 1151.417264 |
| rs11668344 | A | G | 0.3597 | -0.4469 | 0.0126 | 2.31E-277 | 20397.4811 |
| rs299168 | G | A | 0.0963 | 0.2382 | 0.0208 | 2.80E-30 | 2007.998579 |
| rs2867235 | C | T | 0.3648 | -0.0726 | 0.0126 | 8.02E-09 | 492.9691933 |
| rs6054257 | G | A | 0.7951 | 0.1427 | 0.0156 | 5.74E-20 | 1344.689097 |
| rs16991615 | G | A | 0.0625 | 1.063 | 0.0252 | 0 | 30727.44752 |
| rs746748 | C | T | 0.0653 | 0.219 | 0.0247 | 7.56E-19 | 1185.612744 |
| rs11699793 | C | T | 0.1028 | -0.1226 | 0.0199 | 6.96E-10 | 559.7424041 |
| rs483508 | C | T | 0.3847 | 0.1173 | 0.0125 | 6.84E-21 | 1319.964849 |
| rs7266248 | A | G | 0.1986 | 0.1146 | 0.0151 | 3.62E-14 | 845.1548034 |
| rs7347500 | G | A | 0.2149 | -0.233 | 0.0151 | 9.32E-54 | 3756.83353 |
| rs6122488 | G | A | 0.5879 | -0.0768 | 0.0124 | 6.40E-10 | 577.0196054 |
| rs2834747 | T | G | 0.2902 | -0.0928 | 0.0134 | 4.89E-12 | 716.7900376 |
| rs1554934 | A | G | 0.3079 | -0.0756 | 0.0132 | 1.06E-08 | 491.5869829 |
| rs111724290 | G | A | 0.0404 | 0.2833 | 0.0315 | 2.54E-19 | 1260.650949 |
| rs139906882 | C | T | 0.0125 | 0.4825 | 0.0615 | 4.16E-15 | 1163.76179 |
| rs138457 | T | C | 0.6349 | 0.1776 | 0.0126 | 7.33E-45 | 2987.580981 |
| rs11705686 | C | A | 0.1618 | -0.0963 | 0.0166 | 5.99E-09 | 507.6818385 |
| rs2885255 | G | C | 0.4024 | -0.0709 | 0.0123 | 8.60E-09 | 487.9005901 |
